# Molecular Characterization of *Sec2* Loci in Wheat—*Secale africanum* Derivatives Demonstrates Genomic Divergence of *Secale* Species

**DOI:** 10.3390/ijms16048324

**Published:** 2015-04-14

**Authors:** Guangrong Li, Hongjun Zhang, Li Zhou, Dan Gao, Mengping Lei, Jie Zhang, Zujun Yang

**Affiliations:** School of Life Science and Technology, University of Electronic Science and Technology of China, Chengdu 610054, China; E-Mails: ligr28@uestc.edu.cn (G.L.); hongjunzh01@sina.com (H.Z.); zzllttmm@sina.com (L.Z.); gaodangaodan2010@163.com (D.G.); leimengping2008@163.com (M.L.); christine219@163.com (J.Z.)

**Keywords:** wheat, *Secale africanum*, genome evolution, *Sec2*, fluorescence *in situ* hybridization

## Abstract

The unique 75 K γ-secalins encoded by *Sec2* loci in *Secale* species is composed of almost half rye storage proteins. The chromosomal location of *Sec2* loci in wild *Secale* species, *Secale africanum*, was carried out by the wheat—*S. africanum* derivatives, which were identified by genomic *in situ* hybridization and multi-color fluorescence *in situ* hybridization. The *Sec2* gene-specific PCR analysis indicated that the *S. cereale Sec2* was located onchromosome 2R, while the *S. africanum Sec2* was localized on chromosome 6R^afr^ of *S. africanum*. A total of 38 *Sec2* gene sequences were isolated from *S. africanum*, *S. cereale* and *S. sylvestre* by PCR-based cloning. Phylogenetic analysis showed that *S. africanum Sec2* diverged from *S. cereale Sec2* approximately 2–3 million years ago. The illegitimate recombination of chromosome 2R–6R involving the *Sec2* loci region may accelerate sequence variation during evolutionary process from wild to cultivated *Secale* species.

## 1. Introduction

The genus *Secale* includes cultivated rye and 4 to 11 wild species depending on the criteria used for species definition [1,2]. Morphological, biochemical and molecular cytogenetic evidence supported that the wild perennial rye species are considered to be the progenitor of cultivated rye (*Secale cereale* L.) [2,3]. Within the genus *Secale*, several translocations and structural changes in chromosomes have been confirmed by genetic and genomic analysis, and they were thought to have played an important role in speciation [4,5,6,7]. Cultivated rye serves as a valuable source of potentially useful genes for wheat improvement [6]. The wild species of rye are also valuable sources for wheat breeding [2]. As an endangered wild species of *Secale*, *Secale africanum* Stapf. was reported to possess less heterochromatin than *S. cereale* [8,9,10,11,12,13]. To introduce novel disease resistance genes from *S. africanum* into common wheat, an amphiploid from the *Triticum turgidum*—*S. africanum* intergeneric hybrid was crossed with cultivated wheat [14,15], and a large number of wheat—*S. africanum* introgression lines were developed [10]. Recently, *S. africanum* chromosomes 1R^afr^, 2R^afr^ and 6R^afr^ were transferred into a cultivated wheat background [12,13,16]. These chromosomes are novel sources to compare the individual chromosome evolution between cultivated and wild *Secale* species.

Secalin proteins are a prolamin (alcohol-soluble) group of seed storage proteins present in rye grain. These proteins can be classified into four major groups: (I) the high-molecular-weight (HMW) secalins; (II) the Mr 40 K γ-secalins; (III) the Mr 75 K γ-secalins; and (IV) the ω-secalins [17]. The 75 K γ-secalins of rye are the most abundant group of secalins, which are present in endosperm with aggregates stabilized by intra-/inter-molecular disulfide bonds [18,19]. The 75 K γ-secalins are encoded by locus *Sec2*, which represents a unique family of 75 K γ-secalins that does not have analogues in other cereals [20]. The *Sec2* locus is found on chromosome 2R in cultivated rye (*S. cereale*) or 6R in wild perennial rye (*S. montanum*) [21,22,23,24]. The analysis of the wheat—*Secale* translocation lines indicated that *Sec2* increased polymeric glutenin, which could potentially provide superior dough properties and increase protein levels in the endosperm [23]. However, there is little sequence information for paralogous genes within the *Sec2* in different rye species [25,26].

In the present study, we isolated the *Sec2* loci from newly developed wheat—*S. africanum* chromosome substitution lines and compared the *Sec2* loci between wild and cultivated *Secale*, with the aim to reveal the sequences evolution and chromosome rearrangement in *Secale* genomes.

## 2. Results

### 2.1. Identification of Wheat—S. africanum Amphiploid and Substitution

**Figure 1:**
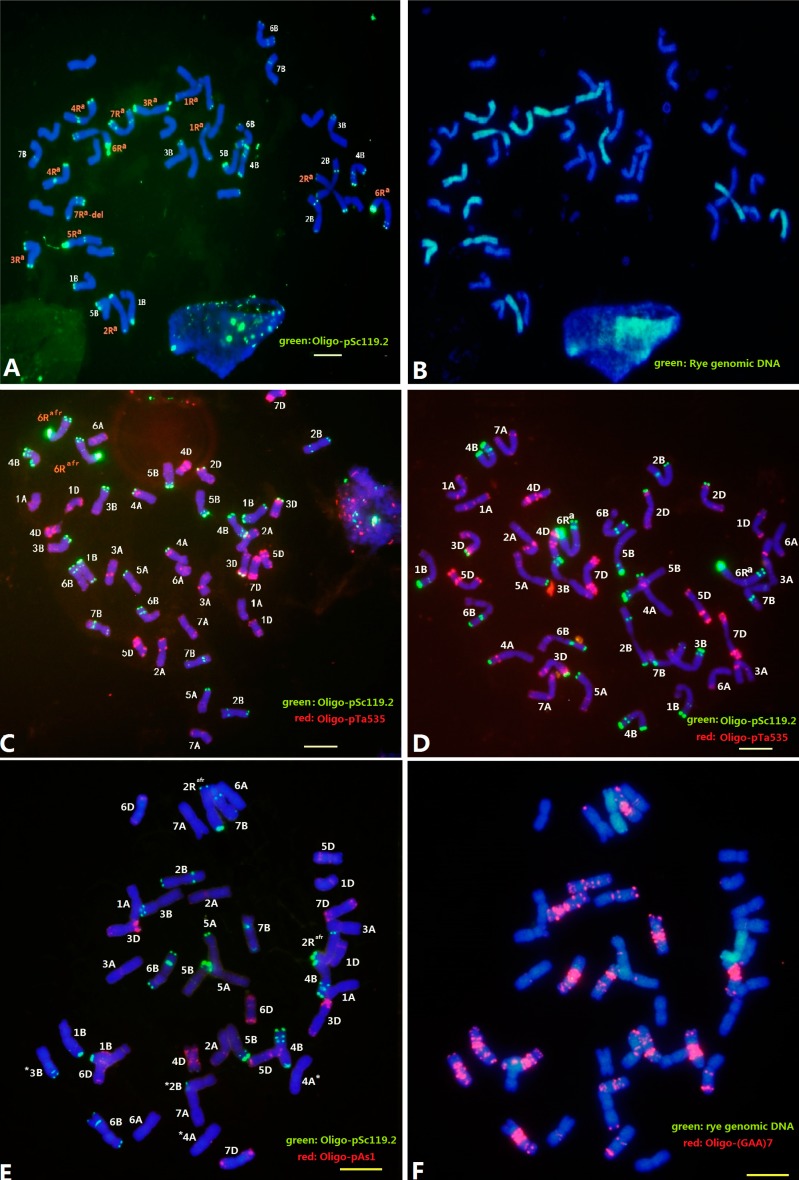
FISH and genomic *in situ* hybridization of *T. durum*—*S. africanum* amphiploid (**A**,**B**); *T. aestivum*—*S. africanum* chromosome 6R^afr^ (6D) substitution lines HH-41 (**C**); Mn512 (**D**); and 2R^afr^ (2D) substitution line LF34 (**E**,**F**). The bar is 10 μm.

With the combination of fluorescence *in situ* hybridization (FISH) probed by repetitive sequences pSc119.2 and genomic *in situ* hybridization (GISH) using *S. cereale* cv. JZHM genomic DNA as the probe, we are able to determine that the *S. africanum* chromatin introgressed in the wheat (*Triticum aestivum*) background. All *S. africanum* chromosomes were identified in *T. durum*—*S. africanum* amphiploid YF-1 (Figure 1A,B). The *S. africanum* chromosome 6R^afr^ displayed a strong FISH signal along the telomeric region on about 1/3 of the short arms, where two bands were located in the terminal and sub-terminal region of long arms. Some faint FISH signals also appeared close to the centromeric region. FISH with probes of Oligo-pTa535 and Oligo-pSc119.2 revealed that the *T. aestivum*—*S. africanum*-derived lines HH-41 and Mn512 were chromosome 6R^afr^ (6D) substitution lines (Figure 1C,D). The chromosome 2R^afr^ was shown by the pSc119.2 signal at both ends in YF-1. FISH probed with Oligo-pAs1, Oligo-pSc119.2 and Oligo-(GAA)_7_ and sequential GISH also confirmed that the *T. aestivum*—*S. africanum*-derived line LF34 was a chromosome 2R^afr^ (2D) substitution line (Figure 1E,F). We found that the banding patterns of *S. africanum* chromosomes 2R^afr^ was the absence of any telomeric region as compared with the YF-1. The results showed that *S. africanum* chromosomes 6R^afr^ and 2R^afr^ displayed a clear FISH banding pattern, and thus, they can easily be distinguished in the wheat background.

### 2.2. Localization of Sec2 in the S. africanum Chromosome

**Figure 2:**
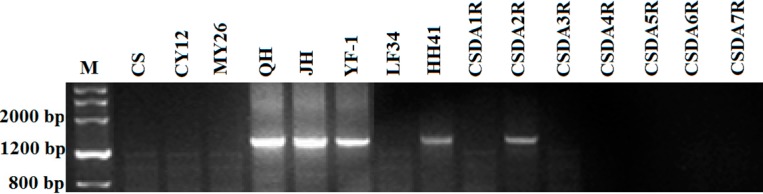
PCR patterns of *Sec2* for wheat lines (CS, CY12 and MY26), *S. africanum*, *S. cereale* cv. JZHM, wheat—*S. africanum* amphiploid (YF-1), 2R^afr^ (2D) substitution (LF34), 6R^afr^ (6D) substitution (HH41) and CS—*S. cereale* cv. Imperial addition lines (CSDA1R to CSDA7R).

In order to localize the *Sec2* (*Gli-R2*) locus encoding 75 K γ-secalin proteins to the specific *Secale* chromosome, the reported *Sec2*-specific PCR primer pairs were used [24]. The genomic DNA isolated from wheat lines CS, CY12, MY26, *S. cereale* cv. JZHM (JH), *S. cereale* cv. QLHM (QH), CS—*S. cereale* cv. Imperial addition lines (CSDA1R to CSDA7R), *T. durum*—*S. africanum* amphiploid YF-1 and wheat—*S. africanum* 6R^afr^ (6D) substitution line HH41 were amplified by the *Sec2* primer set (Figure 2). The *Sec2* gene on *S. cereale* chromosome 2R is clearly visible, since it can be amplified in CSDA2R. However, there was no amplification of *Sec2* in wheat—*S. africanum* 2R^afr^ derivative line LF34, while in wheat—*S. africanum* 6R^afr^ substitution line HH41, about a 1200–1500 bp product was amplified with *Sec2*-specific primers. The PCR results suggested that the *Sec2* loci is located on *S. africanum* chromosome 6R^afr^.

### 2.3. The Sequence Homology of Sec2 Genes

Genomic DNA was isolated from YF-1 and HH41 representing *S. africanum*, CSDA2R representing *S. cereale*, together with *S. sylvestre*. Genomic DNA was used as templates, and PCR amplification for the complete sequences of *Sec2* genes was conducted using the *Sec2* complete sequence primer [26]. The PCR products were cloned in vector pT7 Blue R-Vector and sequenced. A total of 38 unique clones, including nine from YF-1, 11 from HH41, nine from CSDA2R and nine from *S. sylvestre*, were sequenced. All sequences were deposited into GenBank under the Accession Numbers JX877738 to JX877776 (Table 1). The nucleotide sequences comparison of the entire sequence showed a high degree of homology with other *Sec2* sequences. Sequence prediction indicated that 27 of 38 sequences included complete open reading frames (ORF) of genes. The rest of the 11 sequences structurally similar to the full-ORF genes were pseudogenes, because they contained a typical in-frame premature stop codon resulting from the transition of T by C at the first base of the glutamine codon (CAA, CAG), either at repetitive central domains or *C*-terminal domains. As shown in Table 1, the *S. africanum Sec2* gene sequences contained 55% (11 of 20) pseudogenes, while no pseudogenes in *S. sylvestre Sec2* genes were found.

**Table 1:** The *Sec2* sequences isolated from wheat—*S. africanum* derivatives, *S. cereale* and *S. sylvestre*.

Materials	Full-ORF	Pseudogenes
CSDA2R	JX877767 (1434 bp, 54.19 kD), JX877768 (1416 bp, 53.58 kD), JX877769 (1416 bp, 53.42 kD), JX877770 (1416 bp, 53.58 kD), JX877771 (1395 bp, 52.64 kD), JX877772 (1215 bp, 45.73 kD), JX877773 (1416 bp, 53.58 kD), JX877774 (1434 bp, 54.22 kD), JX877775 (1395 bp, 52.64 kD), JX877776 (1395 bp, 52.64 kD)	
S. sylvestre	JX877738 (1503 bp, 56.73 kD), JX877739 (1503 bp, 56.71 kD), JX877740 (1503 bp, 56.71 kD), JX877741 (1503 bp, 56.71 kD), JX877742 (1203 bp, 45.03 kD), JX877743 (1503 bp, 56.70 kD), JX877744 (1503 bp, 56.58 kD), JX877745 (1503 bp, 56.71 kD), JX877746 (1503 bp, 56.73 kD)	
YF-1	JX877747 (1428 bp, 53.98 kD), JX877748 (1428 bp, 54.03 kD), JX877749 (1260 bp, 47.38 kD), JX877750 (1260 bp, 47.38 kD)	JX877751(1371 bp), JX877752(1356 bp), JX877753 (1356 bp), JX877754 (1356 bp), JX877755 (1356 bp)
HH41	JX877756 (1428 bp, 54.39 kD), JX877757 (1464 bp, 55.23 kD), JX877758 (1428 bp, 53.90 kD), JX877759 (1452 bp, 54.64 kD), JX877760 (1311 bp, 49.04 kD)	JX877761 (1320 bp), JX877762( 1332 bp), JX877763 (1332 bp), JX877764 (1332 bp), JX877765 (1320 bp), JX877766 (1332 bp)

### 2.4. The Amino Acid Sequences of Sec2 Genes

**Figure 3:**
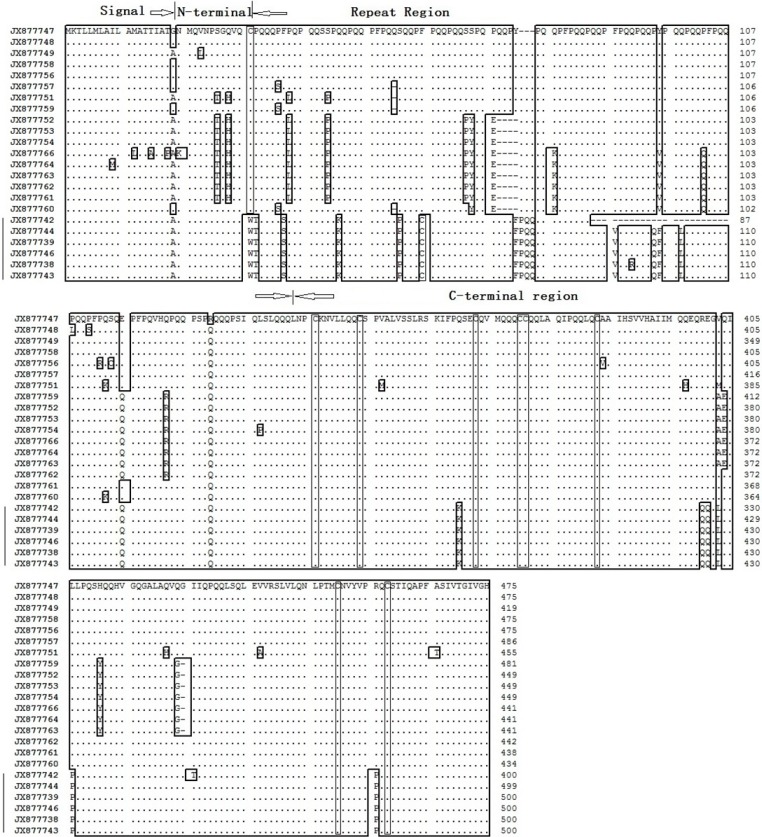
Alignment of deduced amino acid sequence *N*- and *C*-terminal regions of *S africanum Sec2* and *S. sylvestre Sec2*. The cysteine residues are boxed. *S. sylvestre Sec2* is marked by a left line*.*

The general structure of *Sec2* protein consists of four main structural domains, including a conservative signal peptide with 19 amino acids, a steady short *N*-terminal region with 12 amino acids containing a cysteine, a repetitive domain and a conserved *C*-terminal domain with 143 amino acids, including seven or eight cysteines [24]. On the basis of the deduced amino acid sequence of the *Secale Sec2* genes, the 26 complete ORFs represented a presumptive mature protein with 241–305 residues and a calculated molecular weight of 47.3–56.7 kD (Table 1). The alignment of the amino acid sequences of *Sec2* genes indicated that all *Sec2* genes contained eight conserved cysteine residues at the end of the *C*-terminal region (Figure 3). The *S. africanum*-derived *Sec2* sequences displayed a cysteine at the *C*-terminal region, while the *S. sylvestre Sec2* sequences showed an extra cysteine at repetitive regions, but a lack of the cysteine at the *C*-terminal region. The extra cysteine was possibly due to a point mutation (C to G), which makes TGC (encoded C) from TCC (encoded S).

### 2.5. Phylogenetic Tree and the Evolutionary Analyses

**Figure 4:**
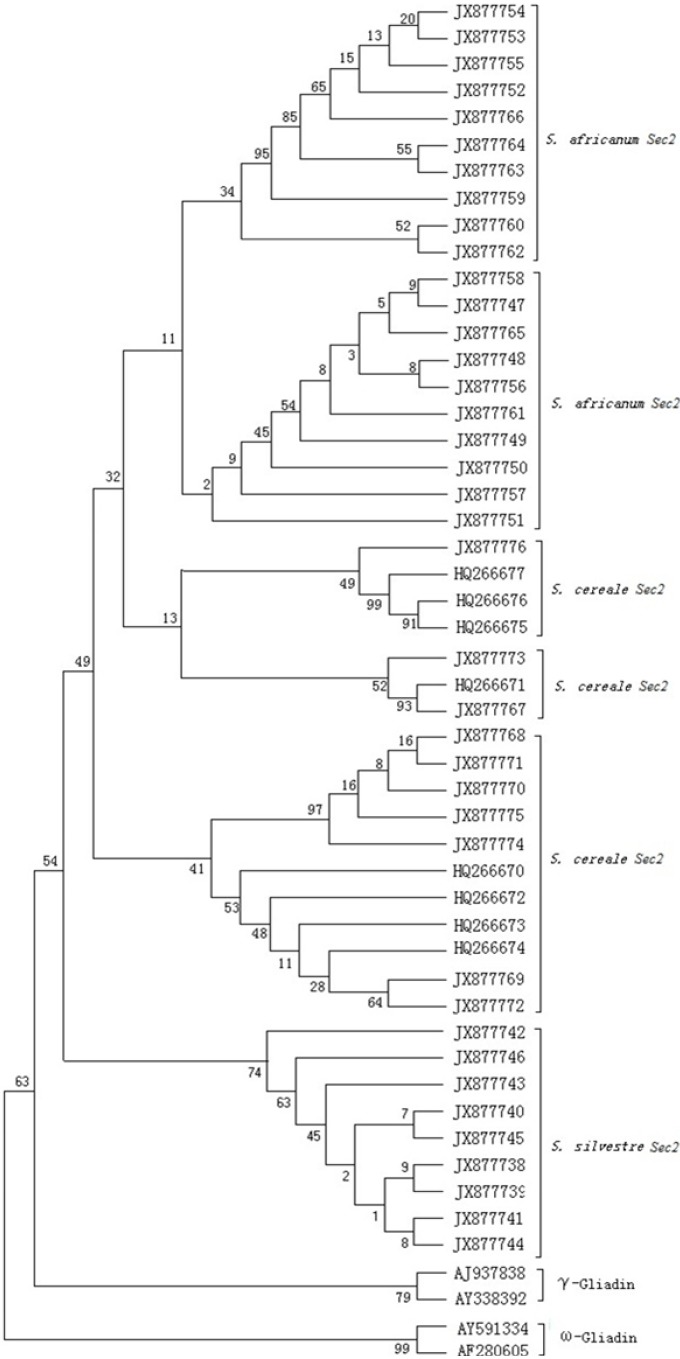
Phylogenetic tree generated from the *Sec2* gene family from *Secale* and the control gliadin of wheat by MEGA4 using the neighbor-joining (NJ) method. Numbers above the branches show bootstrap frequencies based on 1000 replicates.

Sequence comparison was performed among the *Secale Sec2* genes to understand the relatedness and the divergent time by construction of phylogenetic trees. In addition to the nucleotide sequence of the other reported eight *Secale Sec2* alleles, four γ-gliadin and ω-gliadin sequences from wheat were included as the outgroup. As shown in Figure 4, the phylogenetic tree indicated that the sequences from wheat were clearly separated from those from *Secale*. The gliadin genes from *S. africanum Sec2* sequences and *S. sylvestre Sec2* sequences were clustered in one group, while the genes from *S. cereale* were clustered into different groups. The number of polymorphic sites (*S*) and nucleotide diversity (π) among *Sec2* sequences of *S. sylvestre*, *S. africanum* and *S. cereale* were calculated. Total *S. cereale Sec2* with *S* = 230 and π = 0.0553 was shown to be significantly higher than those of *S. africanum Sec2* with *S* = 79 and π = 0.0399, while the *S. sylvestre Sec2* sequences exhibited the lowest variation with *S* = 32 and π = 0.0016*.* This fact suggests that the *Sec2* genes in *S. cereale* exhibited higher diversity than those in *S. africanum* and *S. sylvestre*. Based on the evolutionary rate of 6.5 × 10^−9^ of cereal prolamin genes in wheat and its relatives [27,28], we found that the divergence of *S. sylvestre Sec2* gene sequences spans around four million years (Mya), while *S. cereale* and *S. africanum Sec2* genes separated approximately 2–3 Mya.

## 3. Discussion

Studies indicated that cultivated rye evolved from wild perennial rye through the appearance of annual weedy forms [1,9]. The wild rye, *S. africanum*, is considered to be an ancient species of *S. cereale*. Devos *et al*. [6] compared the molecular marker homologous groups between wheat and rye and found that the chromosomal rearrangement occurred between wheat and rye chromosomes. Recently, Martis *et al.* [7] used high-throughput genome sequences to compare the *Secale* with barley, *Brachypodium* and rice genomes. They revealed that the modern rye genomes exhibited significant chromosomal rearrangements compared to their ancestral genomes. However, the studies had difficulty revealing the genomic variation and recombination during the evolution or domestication between wild and cultivated *Secale* species. In order to interpret the divergence and evolution among *Secale* species by comparing the corresponding chromosomes or chromosome regions, we produced and identified several wheat—*S. africanum* derivative lines, including the addition, substitution or translocation lines [12,13,14]. These lines will allow the comparison of different *Secale* genomes based on cytogenetic, biochemical and molecular evidence.

More precise evidence has been accumulated over the past decade by the establishment of the chromosomal locations of an array of biochemical and molecular markers, which was used to interpret the origins of the genetic recombination in rye chromosomes [6,7,13]. A group of endosperm storage proteins were reported to be located on chromosome 2R in *S. cereale* and then located on chromosome 6R^mon^ of *S. montanum* [17]. The loci of *Gli-2* (*Sec2*) are located on 6AS, 6BS and 6DS in wheat, 2RS in *S. cereale* and 6R^mon^ in *S. montanum*. This indicated a translocation between part of the short arm of a group 2 chromosome and part of the short arm of a group 6 chromosome (2S/6S translocation) in the genome of *S. cereale*, relative to those of wheat and *S. montanum* [22]. In the present study, we found that the *Sec2*-specific markers were assigned to *S. africanum* chromosome 6R^afr^, not 2R^afr^, which supports the theory that translocations have taken place in the ancestor of cultivated rye [6,7].

The *Sec2* (*Gli-R2*) locus encoding 75 K γ-secalin proteins is located relatively close to the telomeric C-band on the short arm of *S. cereale* chromosome 2R [24]. A genomic DNA clone coding for a rye secalin gene (gSec2A) was isolated from a wheat-rye translocation line carrying the 2RS.2BL chromosome [25]. Recently, Wang *et al*. [29] identified 59 *Sec2* sequences from a cultivated rye and derivative lines after crossing with bread wheat. Chen *et al*. [26] isolated four *Sec2* gene sequences from each of *S. cereale*, *S. vavilovii*, *S. sylvestre* and *S. strictum* using a PCR-based strategy, implying that the *Sec2* genes are conserved in genus *Secale*. Our studies reported new *Sec2* gene sequences from *S. sylvestre* and *S. africanum* in wheat—*S. africanum* amphiploid and substitution lines and found the novel variations at the nucleotide and amino acid level in the *Sec2* gene loci. It will be interesting for further studies to be done on the genetics and interaction between wheat and *Secale* gliadin proteins for wheat and triticale breeding for quality improvement.

Since the unique family of the 75 K γ-secalins *Sec2* loci does not have analogues in other cereals [20], the estimation of seed protein evolutionary divergence time can be performed after species become reproductively isolated. Therefore, molecular dating can estimate the divergence of a particular genetic locus, but not the divergence of species [30]. The estimation of *S. africanum Sec2* diverged from *S. cereale Sec2* approximately 2–3 million years ago (Mya). It can also be interpreted that *S. africanum* is one of the perennial *Secale*, with restricted geographic distribution in South Africa, today on the verge of extinction [8,31]. Rye and wheat diverged 7 Mya, and both lineages and the barley lineage diverged from a common Triticeae ancestor around 11 Mya [32]. The complicated chromosomal rearrangements of the rye genome might have occurred during evolution less than 2–3 Mya. Higher diversity of *S. cereale Sec2* gene sequences in 2R than *S. africanum Sec2* in 6R^afr^ was possibly a result of the location of the 2RS terminal region in a hotspot region in the cereal genome. It will be able to address the issue of a correspondence between the chromosomal gaps and rearrangements for the adaptation of the wild *Secale* species.

Recently, the occurrence of chromosome structure variation induced by the *S. cereale* chromosome in wheat-rye derivatives was observed [33]. In the self-progeny of the 6R (6D) substitution line, several kinds of altered chromosomes were also discovered [34]. It is likely that the wheat—*S. cereale* chromosome 6R monosomic addition line could induce the alterations of wheat chromosomes and the abnormal behavior of wheat chromosomes during mitosis. However, in the present study, no wheat chromosome structural changes were observed in the wheat—*S. africanum* 6R^afr^ (6D) substitution lines. We assumed that the *S. cereale* chromosome 6R might have lost some factors that affect the stability of the wheat background, while the ancestry of *S. africanum* contained chromosome 6R^afr^.

## 4. Experimental Section

### 4.1. Plant Materials

*Secale africanum*, *S. sylvestre* and *S. vavilovii* were kindly provided by Perry Gustafson of the University of Missouri, Columbia, MO, USA. The *T. durum*—*S. africanum* amphiploid (YF-1), *S. cereale* cv. Jinzhouheimai (JZHM) and wheat cultivars Mianyang11 (MY11), Chuanyu12 (CY12) and Mianyang 26 (MY26) are maintained in our laboratory. Chinese spring—*S. cereale* Imperial rye addition lines (CSDA1R to CSDA7R) were obtained from Bernd Friebe, Wheat Genetic and Genomic Resources Center, Kansas State University, Manhattan, KS, USA. The *S. africanum* chromosome 2R^afr^ (2D) substitution line LF24 and chromosome 6R^afr^ (6D) substitution line HH41 were selected from the F_7_ generation of the cross between wheat cultivars Mianyang11 (MY11) and YF-1.

### 4.2. Genomic in Situ Hybridization and Fluorescence in Situ Hybridization

Seedlings were grown in petri dishes, and the root tips of about 2 cm were collected and pretreated in water at 0 °C for 24 h and fixed in ethanol-acetic acid (3:1) for 1 week. Root-tip squashes and chromosome preparation were done according to Yang *et al*. [10]. For genomic *in situ* hybridization (GISH) analysis, the sheared genomic DNA of *S. cereale* cv. JZHM was labeled with Alexa Fluor-488-5-dUTP (Vector Laboratories, Burlingame, CA, USA), and sheared genomic DNA of Chinese spring wheat was used as the blocking. The GISH protocols are from Yang *et al*. [10]. The multi-color fluorescence *in situ* hybridization (mcFISH) by the oligonucleotide probes representing the repetitive sequences was used for identifying the wheat chromosomes according to the recent study of Tang *et al*. [35]. Oligo-pSc119.2-1 and Oligo-(GAA)_7_ repeats were end-labelled with 6-carboxyfluorescein (6-FAM) for green signals, and the Oligo-pAs1 and Oligo-pTa535-1 were labeled with 6-carboxytetramethylrhodamine (Tamra) for red signals. Oligonucleotide probes were synthesized by Shanghai Invitrogen Biotechnology Co., Ltd. (Shanghai, China). The synthesized probes and the FISH hybridization process was according to Tang *et al.* [35]. The slides were mounted in 4',6'-diamidino-2-phenylindole (DAPI) dissolved in Vectrashield^®^ antifade solution (Vector Laboratories, Burlingame, CA, USA). Microphotographs of GISH and FISH chromosomes were taken with an Olympus BX-51 microscope using a DP-70 CCD camera.

### 4.3. Primer Design, PCR Cloning and Sequencing

Total genomic DNA was isolated from young leaves as described by Li *et al*. [36]. The DNA concentration was determined using a Sizhumen DNA-protein photometer and also by comparison with a known lambda DNA standard on a 1% agarose gel. The *Sec2*-specific PCR primer pair synthesis and PCR protocol followed that of Chen *et al*. [26]. The target genes amplified by PCR were excised from 1.0% agarose gels and purified using a gel extraction kit (Qiagen, Valencia, CA, USA). The purified products were ligated into the pT7 Blue R-Vector using T4 ligase and then introduced into *Escherichia coli* DH5α by heat shock transformation. Nucleotide sequencing was performed on a polyacrylamide gel with the ABI prism 377 sequencer (Perkin Elmer) as an automated fluorescent sequencing system.

### 4.4. Phylogenetic Analyses

The controlled wheat gliadin used as comparison controls was obtained from the NCBI website [37] The *Sec2* gene sequences from the wheat—*S. africanum* 6R^afr^ substitution line, CSDA2R and *S. sylvestre* sequences cloned here were analyzed by the ORF finder program at the NCBI network service [37]. Sequences were aligned using BioEdit software. The sequences HQ266670–HQ266677 of *S. cereale Sec2* genes published by Wang *et al*. [29] were also included in our study. All DNA sequences were aligned using ClustalW Version 1.8 [38]. Multiple alignment parameters were scored up to 12 for the gap opening penalty and 0.1 for the gap extension penalty. Alignments were confirmed manually using sequential pairwise comparisons. MEGA4 was used for calculating pairwise sequence divergences and nucleotide compositions and for performing neighbor-joining (NJ) analyses [39]. The phylogenetic tree was linearized assuming equal evolutionary rates in all lineages and was drawn to scale, with branch lengths in the same units as those of the evolutionary distances used to infer the phylogenetic tree. A consensus tree was generated using 1000 bootstrap replicates. DnaSP Version 5.0 [40] was used to compare the nucleotide diversity [41] of the *Sec2* DNA polymorphism among *Secale* species.

## References

[1] Vavilov N.I. (1926). Studies on the origin of cultivated plants. Bull. Appl. Bot. Plant Breed..

[2] Tang Z.X., Ross K., Ren Z.L., Yang Z.J., Zhang H.Y., Chikmawati T., Miftahudin, Gustafson J.P., Kole C. (2011). Wealth of wild species: Role in plant genome elucidation and improvement—*Secale*. Wild Crop Relatives: Genomic and Breeding Resources Cereals.

[3] Cuadrado A., Jouve N. (2002). Evolutionary trends of different repetitive DNA sequences during speciation in the genus *Secale*. J. Hered..

[4] Riley R. (1955). The cytogenetics of the differences between some *Secale* species. J. Agric. Sci..

[5] Stutz H.C. (1972). On the origin of cultivated rye. Am. J. Bot..

[6] Devos K.M., Atkinson M.D., Chinoy C.N., Francis H.A., Harcourt R.L., Koebner R.M.D., Liu C.J., Masojc P., Xie D.X., Gale M.D. (1993). Chromosomal rearrangements in the rye genome relative to that of wheat. Theor. Appl. Genet..

[7] Martis M.M., Zhou R., Haseneyer G., Schmutzer T., Vrána J., Kubaláková M., König S., Kugler K.G., Scholz U., Hackauf B. (2013). Reticulate evolution of the rye genome. Plant Cell.

[8] Hammer K., Khoshbakht K. (2005). Towards a “red list” for crop plant species. Genet. Resour. Crop. Evol..

[9] Lukaszewski A.J., Gustafson J.P., Janick J. (1987). Cytogenetics of triticale. Plant Breeding Reviews.

[10] Yang Z.J., Li G.R., Jia J.Q., Zeng X., Lei M.P., Zeng Z.X., Zhang T., Ren Z.L. (2009). Molecular cytogenetic characterization of wheat—*Secale africanum* amphiploids and derived introgression lines with stripe rust resistance. Euphytica.

[11] Lei M.P., Li G.R., Liu C., Yang Z.J. (2012). Characterization of new wheat—*Secale africanum* derivatives reveals evolutionary aspects of chromosome 1R in rye. Genome.

[12] Lei M.P., Li G.R., Zhang S.F., Liu C., Yang Z.J. (2011). Molecular cytogenetic characterization of a new wheat—*Secale africanum* 2R^a^ (2D) substitution line for resistant to stripe rust. J. Genet..

[13] Lei M.P., Li G.R., Zhou L., Li C.H., Liu C., Yang Z.J. (2013). Identification of wheat—*Secale africanum* chromosome 2R^afr^ introgression lines with novel disease resistance and agronomic characteristics. Euphytica.

[14] Yang Z.J., Li G.R., Ren Z.L. (2000). Identification of amphiploid between *Triticum durum* cv. Ailanmai native to Sichuan, China and *Secale africanum*. Wheat Inform. Serv..

[15] Yang Z.J., Li G.R., Ren Z.L. (2001). Identification of *Triticum durum*-*Secale africanum* amphiploid and its crossability with common wheat.. J. Genet. Breed..

[16] Lei M.P., Li G.R., Liu C., Yang Z.J. (2013). Molecular cytogenetic characterization of a *Triticum durum*—*Secale africanum* substitution line for resistance to stripe rust (*Puccinia striiformis* Eriks. f. sp. *tritici*). J. Agric. Biotechnol..

[17] Shewry P.R., Parmar S., Miller T.E. (1985). Chromosomal location of the structural genes for the Mr 75,000 γ-secalins in *Secale montanum* Guss: Evidence for a translocation involving chromosomes 2R and 6R in cultivated rye (*Secale. cereale* L.). Heredity.

[18] Shewry P.R., Field J.M. (1982). The purification and characterization of two groups of storage proteins (secalins) from rye (*Secale cereale* L.). J. Exp. Bot..

[19] Shewry P.R., Kreis M., Burgess S.R., Parmer S., Miflin B.J. (1983). The synthesis and deposition of the prolamin storage proteins (secalins) of rye. Planta.

[20] Gellrich C., Schieberle P., Wieser H. (2005). Studies of partial amino acid sequences of γ-40k secalins of rye. Cereal Chem..

[21] Lawrence G.J., Shepherd K.W. (1981). Chromosomal location of genes controlling seed proteins in species related to wheat. Theor. Appl. Genet..

[22] Shewry P.R., Bradberry D., Franklin J., White R.P. (1984). The chromosomal locations and linkage relationships of the structural genes for the prolamin storage proteins (secalins) of rye. Theor. Appl. Genet..

[23] Shewry P.R., Miflin B.J., Kasarda D.D. (1984). The structural and evolutionary relationships of the prolamin storage proteins of barley, rye and wheat. Philos. Trans. R. Soc. Lond. B Biol. Sci..

[24] Hull G., Sabelli P.A., Shewry P.R. (1992). Restriction fragment analysis of the secalin loci of rye. Biochem. Genet..

[25] Murray F.R., Skerritt J.H., Appels R. (2001). A gene from the *Sec2* (Gli-R2) locus of a wheat 2RS.2BL chromosomal translocation line. Theor. Appl. Genet..

[26] Chen Q.J., Yuan Z.W., Zhang L.Q., Yan Z.H., Xiang Z.G., Wan Y.F., Zheng Y.L., Liu D.C. (2008). Molecular characterization and comparative analysis of four new genes from *Sec2* locus encoding 75K γ-secalin of rye species. J. Cereal Sci..

[27] Allaby R.G., Banerjee M., Brown T.A. (1999). Evolution of the high molecular weight glutenin loci of the A, B, D, and G genomes of wheat. Genome.

[28] Gaut B.S., Morton B.R., McCraig B.C., Clegg M.T. (1996). Substitution rate comparisons between grasses and palms: Synonymous rate differences at the nuclear gene *Adh* parallel rate differences at the plastid gene *rbcL*. Proc. Natl. Acad. Sci. USA.

[29] Wang Y., Zhang B., Liu B., Zhang H., Liu D. (2012). Structure and evolutionary relationships among paralogous genes within the *Sec2* locus in rye. J. Cereal Sci..

[30] Middleton C.P., Senerchia N., Stein N., Akhunov E.D., Keller B., Wicker T., Kilian B. (2014). Sequencing of chloroplast genomes from wheat, barley, rye and their relatives provides a detailed insight into the evolution of the Triticeae tribe. PLoS ONE.

[31] Jenabi T., Saeidi H., Rahiminejad M.R. (2011). Biodiversity of *Secale strictum* in Iran measured using microsatellites. Genet. Resour. Crop. Evol..

[32] Huang S., Sirikhachornkit A., Faris J.D., Su X., Gill B.S., Haselkorn R., Gornicki P. (2002). Phylogenetic analysis of the acetyl-CoA carboxylase and 3-phosphoglycerate kinase loci in wheat and other grasses. Plant Mol. Biol..

[33] Fu S., Lv Z., Guo X., Zhang X., Han F. (2013). Alteration of terminal heterochromatin and chromosome rearrangements in derivatives of wheat-rye hybrids. J. Genet. Genomics.

[34] Fu S., Yang M., Fei Y., Tan F., Ren Z., Yan B., Zhang H., Tang Z. (2013). Alterations and abnormal mitosis of wheat chromosomes induced by wheat-rye monosomic addition lines. PLoS ONE.

[35] Tang Z., Yang Z., Fu S. (2014). Oligonucleotides replacing the roles of repetitive sequences pAs1, pSc119.2, pTa-535, pTa71, CCS1, and pAWRC.1 for FISH analysis. J. Appl. Genet..

[36] Li G., Lang T., Dai G., Li D., Li C., Song X., Yang Z. (2015). Precise identification of two wheat—*Thinopyrum intermedium* substitutions reveals the compensation and rearrangement between wheat and *Thinopyrum* chromosomes. Mol. Breed..

[37] NCBI network service. http://www.ncbi.nlm.nih.gov/.

[38] Thompson J.D., Higgins D.G., Gibson T.J. (1994). CLUSTAL W: Improving the sensitivity of progressive multiple sequence alignment through sequence weighting, position-specific gap penalties and weight matrix choice. Nucleic Acids Res..

[39] Tamura K., Dudley J., Nei M., Kumar S. (2007). MEGA4: Molecular evolutionary genetics analysis (MEGA) software version 4.0. Mol. Biol. Evol..

[40] Librado P., Rozas J. (2009). DnaSP v5: A software for comprehensive analysis of DNA polymorphism data. Bioinformatics.

[41] Nei M. (1987). Molecular Evolutionary Genetics.

